# The Thermal Waters of the Eastern Half of the United States

**Published:** 1915-02

**Authors:** Felix Von Oefele

**Affiliations:** New York; 326 E. 58th St.


### The Thermal Waters of the Eastern Half of the United States.

DR. FELIX VON OEFELE, New York.

In the first paper I spoke of the nitrated low mineralized springs of New England, in the second paper of the silicated low mineralized springs in the same neighborhood. It is not very easy without analysis to recognize the mineral spring character of nitrated or silicated springs, as it is to recognize thermal waters. Every layman is able to classify correctly a thermal water; but the thermal waters are less numerous than cold mineral springs.

As stated in the two former papers, the location of mineral springs is in either lines or groups, but with very few exceptions each time in relation to mountain systems.

There is a very important official bulletin of Rudolph Ruedemann, about the Geology of the Eastern part of the United States; it is a publication of Education Department, Albany 1910 Bulletin. The title of which is "On the symmetric arrangement in the elements of the paleozoic platform of North America." In this bulletin he states there is one paleozoic mass in the Adirondacks, a second such mass in the Appalachian Mountains, a third one, in the state of Wisconsin and a fourth, in the Ozark Mountains. To the north of these four paleozoic islands is a paleozoic Canadian Continent.

As we have learned in the arrangement of nitrated springs, mineral springs are often arranged in lines corresponding to geological faults. Such faults are mostly the result of the geologic arising of some mountain systems in the neighborhood. The magmatic mineral waters rise through these faults and are in many cases still of higher temperature than the surrounding country. In the western mountain systems of younger geologic age, there are very many thermal mineral springs. The older four mentioned paleozoic mountain systems of the east contain only few thermal water springs.

The symmetric arrangement of the four paleozoic masses repeats in four corresponding groups of thermal waters. I have not enough data on hand to determine the exact situation and boundaries of the thermal waters in the center of the state of Wisconsin. Arkansas Hot Springs belongs to the Ozark paleozoic mass; it would also lead too far to enumerate other thermal waters that relate closely to the group of Arkansas Hot Springs. Virginia Hot Springs and many other thermal waters of their vicinity belong to the Appalachian paleozoic masses.

The readers of the Buffalo Medical Journal are more interested in the springs of the State of New York, and in this way for the thermal waters in connection with the Adirondack

Mountains. Yet there are very few readers that would not be astonished by the existence of such thermal springs in the Adirondack Mountains.

I was only able to find the Sands Springs, Mass. and Lebanon Springs, New York; the district there is known as Taconic Mountains with a center point in Berlin Mountain. Berlin Mountain is about situated at the tri-state boundary point of New York, Massachusetts and Vermont. In a balneological sense, this district is insufficiently studied; there are few railroads and few vacation visitors there, the tourists prefer going to the Catskills or to the Adirondacks. Berlin Mountain being out of the way for such trips. These circumstances are the only reason that only two springs in this neighborhood are known and must be classified as thermal springs. An increased accessibility of the Taconic Mountains will surely increase in the near future, the number of natural warm waters. The entire northeast of United States does not contain any natural warm waters, else the spring group of the Taconic Mountains must therefore be called the thermal spring group belonging to the Adirondack Paleozoic System.

Sands Spring, Mass., is a tepid spring of 76° fahr. and Lebanon Spring, New York of 75° fahr. Sand Spring contains 0.0195% of total solids and Lebanon Springs 0.0413%. Sands Spring belongs to the very low mineralized springs and Lebanon Spring to the low mineralized springs of the second degree. Low mineralized thermal waters are very famous throughout Europe, for cure by drinking at the springs; they are called indifferente Thermes or Akratothermes. In Germany they are mostly called Wildbad; there is known Badenweiler in the black forest of Baden 26.40° C and 0.035% total solids, very similar to the mentioned Lebanon Spring in New York; this is recommended for morbus Basedowi; convalescence after acute diseases, residues of pleuritis, for organic or functional diseases, nerve diseases, for chronic women's diseases, for gout, rheumatism and obesity.

A net work of well kept pathways and uneven forests at a bathing cure place enables good results, on diseases of the heart and bloodvessels. The late Professor Liebreich made special studies as to the influence of low mineralized water to the animal skin. A therapeutic influence of Lebanon Spring and Sand Spring for skin diseases then can be imagined to give good results. All mineral waters but especially the thermal waters possess a higher electric conductivity on account of the higher ionization. This increased conductivity is not lost if thermal waters become cold and are again heated, their ionization and conductivity may sometimes stay for years.

If Professor Liebreich has proven the influence of the springs to the animal skin, there must also be an increased influence of thermal waters to the mucus membranes and also in the body's liquid immediately after absorption.

Other European Akratothermes are Krapina-Toeplitz, Tueffers, Teplitz, Tobelbad, Voeslau. It seems to me, this should be sufficient to show how useful Sands Spring and Lebanon Spring and perhaps many others, not yet discovered springs of the Taconic Mountains could be for the medical profession, because they can replace all the mentioned famous European Spring resorts.

326 E. 58th St.

---

**Normal Differential Leucocyte Count.** Sidney R. Miller, Johns Hopkins Hosp. Bull., Oct. 19, 1914, cites the following figures. P.M.N., polymorphenuclear neutrophiles; P.M.E. polymorphonuclear eosinophiles; P.M.B. polymorphonuclear basophiles; S.M. small mononuclears; L.M. large mononuclears; T. transitionals. The author's counts, based on examinations of 50 students, 50,000 leucocytes counted per student, 250 in a smear, are as follows: P.M.N. 60-70%, slightly more than half below 65%. P.M.E. and P.M.B., averaging as in the table. S.M., as for the majority of other reporters, values being slightly lower with Ehrlich stain. L.M. including a lymphocytes of size of P.M.N. or larger and (b) cells with large, round, oval or slightly indented nucleus eccentrically placed and poorly stained, surrounded with considerable amount of protoplasm. the former type, true large lymphocytes 1.16%, both types 8.08%. All types of S.M. and L.M. aggregating 30.3%. Transitionals, 2-4%, results slightly lower with Ehrlich stain, which is better for granules and poorer for nuclei, whereas Wilson's is the opposite and Jenner's more of an average stain.

Average Percentage Values of Leucocytes in Normal Blood.

| | Author | P.M.N. | P.M. E. | P.M. B. | S.M. | L.M. | T. |
|---|---|---|---|---|---|---|---|
| 1. | Pappenheim ...... | 70-75 | 2-4 | 0-1 | 20-22 | 2....... | 6 |
| 2. | Turck ........... | 70-75 | 2-4 | .1-.5 | 22-25 | 1 | 1-4 |
| 3. | Sahli ..........<br>Emerson .......<br>Ehrlich ........ | 70-72 | 2-4 | .5 | 22-25 | 1 | 1-4 |
| 4. | Morawitz ........ | 65-75 | 2-4 | .5 | 20 | 5....... | 7 |
| 5. | Webster ......... | 65-75 | 2-4 | .5 | 20-25 | 3....... | 5 |
| 6. | Wood ........... | 65-75 | 2-4 | .5 | 22-25 | 1 | 2-4 |
| 7. | Krause .......... | 65-70 | 2-3 | .5 | 22-25 | 3....... | 5 |
| 8. | Naegeli .......... | 65-70 | 2-4 | .5 | 22-25 | 3....... | 5 |
| 9. | Brugsch & S...... | 65-70 | 2-4 | .5 | 20-25 | 3....... | 5 |
| 10. | Morris ........... | 65-70 | 2-4 | .5 | 22-25 | 3....... | 5 |
| 11. | Cabot ........... | 60-70 | .5-4 | .1-.5 | 20-40 | 1...... | 10 |
| 12. | Simon ........... | 60-70 | 1-4 | .2-1 | 20-30 | 1....... | 6 |
| 13. | Bunting ......... | 50-60 | .8-4 | .4-1.8 | 30-40 | .6-2 | 6-8 |

